# Short-Term Weight Gain after Tonsillectomy Does Not Lead to Overweight: A Systematic Review

**DOI:** 10.3390/nu16020324

**Published:** 2024-01-22

**Authors:** Pietro Buono, Evelina Maines, Nicolò Azzolini, Roberto Franceschi, Fedi Ludovica, Letizia Leonardi, Luisa Occhiati, Enza Mozzillo, Claudio Maffeis, Marco Marigliano

**Affiliations:** 1 Directorate General of Health, Campania Region, 80131 Naples, Italy; petro.buono2@regione.campania.it; 2 Pediatric Diabetology Unit, Pediatric Department, S. Chiara General Hospital, Azienda Provinciale per i Servizi Sanitari del Trentino, 38122 Trento, Italy; evelina.maines@apss.tn.it (E.M.); nicolo.azzolini@apss.tn.it (N.A.); lleonardi39@gmail.com (L.L.); 3 Department of Translational Medical Science, Section of Pediatrics, Regional Center of Pediatric Diabetes, Federico II University of Naples, 80131 Naples, Italy; ludovicafedi1@gmail.com (F.L.); luisaocchiati@gmail.com (L.O.); 4 Department of Surgery, Dentistry, Pediatrics and Gynecology, Section of Pediatric Diabetes and Metabolism, University and Azienda Ospedaliera Universitaria Integrata of Verona, 37134 Verona, Italy; claudio.maffeis@univr.it (C.M.); marco.marigliano@univr.it (M.M.)

**Keywords:** weight gain, overweight, tonsillectomy

## Abstract

Different studies and systematic reviews have reported weight increase after tonsillectomy. However, the odds of a child being overweight or obese after tonsillectomy were no different than before surgery, according to a few studies. This systematic review aims to analyze the impact of adenotonsillectomy (TA) on weight gain and identify subgroups of children and adolescents at risk of experiencing weight gain. A systematic search included studies published in the last ten years. The PICO framework was used in the selection process, and evidence was assessed using the GRADE system. A total of 26 studies were included, and moderate–high level quality ones showed that children who underwent TA could present an increase in BMI z-score. However, this weight gain was significant in individuals younger than six years old and was considered catch-up growth in underweight subjects at baseline. In contrast, for normal-weight or overweight individuals, TA did not lead to overweight per se. At the same time, diet changes and overfeeding did not have a leading role in weight gain. In conclusion, TA may not be an independent risk factor for unfavorable weight gain in children; however, individuals who were underweight pre-operatively or younger than six years reported more weight gain after TA than expected.

## 1. Introduction

In the last 20 years, the prevalence of overweight and obesity among children and adolescents has increased rapidly. The principal cause is an imbalance between calorie intake and physical activity, with socioenvironmental factors playing a crucial role [1]. 

Tonsillectomy is a standard surgical procedure in the pediatric age, and it is indicated for treating obstructive sleep apnea (OSA) and chronic throat infections. Adenoidectomy is performed for nasal passage obstruction, OSA, recurrent otitis media, and rhinosinusitis. Obesity has been recognized as a risk factor for OSA [2], and children with obesity have more tonsillectomy/adenoidectomy procedures compared with their peers [3,4]. Although tonsillectomy, with or without adenoidectomy, is a relatively safe surgery, postoperative complications have been reported in multiple studies. Among these, post-tonsillectomy hemorrhage is the most common, followed by possible infections, taste disturbances, breathing problems, and dehydration [5]. 

The connection between tonsillectomy and weight gain and growth has been controversial and discussed for decades [6,7]. Weight increase after tonsillectomy has been reported in different studies and the most recent systematic reviews [8,9], but the odds of a child being overweight or obese after tonsillectomy in the next 12–18 months were not different than they were before surgery, according to a few studies [10,11]. Other study designs, methods, baseline weight and age of the subjects, additional indications for tonsillectomy, and outcomes analyzed likely explain the heterogeneity in the literature results.

The mechanisms underlying weight gain after adenotonsillectomy (TA) surgery are still unknown [12]: (i) children with enlarged adenoids and/or tonsils could experience more frequent infections, swallowing problems, dysphagia, or odynophagia, and these disorders can lead to decreased calorie intake; when the tonsils are removed, children can consume a more significant amount of calories [13]; (ii) in children with OSA, intermittent upper airway obstruction during sleep has been thought to increase the work of breathing, and therefore increase energy expenditure at night, and probably after TA, this energy expenditure decreases [9]; (iii) in addition, reduced episodes of OSA after TA could lead to hormonal dysregulation, increasing levels of insulin-like growth factor-1 and insulin growth factor binding protein-3 [14].

The aims of this systematic review are:

(1) to provide an up-to-date summary of the available evidence on the impact of TA on weight gain and eventually increased prevalence of overweight and obesity;
(2) to identify subgroups of children and adolescents at risk of weight gain;
(3) to elucidate mechanisms beyond increased caloric intake that underlie weight gain.

## 2. Materials and Methods

### 2.1. Search Strategy 

We searched electronic databases (Pubmed, EMBASE, The Cochrane Library, Web of Science, Clinicaltrial.gov, International Clinical Trials Registry Platform) for studies published between 1 January 2013, and 1 August 2023. Search terms or “MESH” (Medical Subject Headings) for this systematic review included different combinations: “weight gain” or “overweight”, or “obesity” or “obese”, or “BMI” or “weight” or “unhealthy weight” or “fat” AND “tonsillectomy” or “adenoidectomy” or “adenotonsillectomy”.

We also screened the reference lists of eligible studies to avoid missing any relevant studies.

### 2.2. Criteria for Study Selection 

We conducted a systematic search of the literature according to the PICOS model (Population, Intervention, Comparison, Results, Study design):


**Population**

**Children and Adolescents (1–18 Years) Who Had Undergone Tonsillectomy**
InterventionTonsillectomy with or without adenoidectomyComparisonHealthy control subjectsOutcomesPreoperative and postoperative (up to 24 months after) growth (weight, height, BMI (body mass index), and relative percentiles for age, body composition, metabolic and hormonal changes  and/or comparison with a control groupStudy designRandomized clinical trials (RCTs), observational studies (cohort, case-control, cross-sectional studies), exploratory studies, mix of qualitative and quantitative studies

Inclusion criteria were: (i) study population: children and adolescents (aged 1–18 years) who had undergone tonsillectomy with or without adenoidectomy; (ii) study type: observational studies (cohort, case-control, cross-sectional studies), exploratory studies, a mix of qualitative and quantitative studies; (iii) review articles were excluded, but their reference lists were screened to identify potentially eligible studies; (iv) only published full papers were included, whereas abstracts only were not included; (v) data on weight gain: weight, height, BMI and relative percentiles, metabolic and hormonal changes, before and after tonsillectomy; (vi) publication date: last ten years (2013–2023) to include studies after the last systematic review reported in the literature [9]. 

Exclusion criteria: (i) data available only for adults ≥18 years or subjects with syndromes (Down, Prader Willi, Rohadnet, etc.); (ii) animal data; (iii) case reports; studies with <10 patients who underwent tonsillectomy; (iv) full paper not available; (v) study not yet published; (vi) studies on tonsillectomy not reporting weight variation within 24 months; (vii) languages other than English were not “a priori” exclusion criteria. 

### 2.3. Data Extraction and Management

Using the search strategy, two independent investigators (AN, LO) screened the titles and abstracts of the identified studies for inclusion. Any discrepancies were resolved by consensus or consultation with a third investigator (RF). After abstract selection, four investigators conducted a complete paper analysis (EMA, LL, LF, LO).

The following characteristics were evaluated for each study in the full paper: (i) reference details: authorship(s); published or unpublished; year of publication; period in which the study was conducted; other relevant cited papers; (ii) study characteristics: study design, topic, treatment period, follow-up duration, region; (iii) population characteristics: number of participants who underwent tonsillectomy with or without adenoidectomy, number with OSA, age and demographic data, baseline weight, BMI, fat, waist circumference and other related parameters; comparator characteristics; (iv) methodology: assessment of growth measurements, metabolic and hormone changes, caloric intake, physical exercise; (v) main results: growth pattern, body composition parameters, factors influencing post-operative growth.

### 2.4. Assessment of the Certainty of the Evidence

We used the GRADE approach (Grading of Recommendations Assessment, Development, and Evaluation) to rank the quality of evidence (www.gradeworkinggroup.org, accessed on 4 October 2023) for the included studies. Two authors (EM and RF) independently assessed the certainty of the evidence for each of the outcomes, and MM resolved discrepancies. In case of risk bias in the study design, imprecision of estimates, inconsistency across studies, indirectness of the evidence, and publication bias, the recommended option of decreasing the level of certainty by one or two levels according to the GRADE guidelines was applied [15]. The GRADE approach results in an assessment of the certainty of a body of evidence and allocation to one of four grades: 


**High:**

**Further Research Is Very Unlikely to Change Confidence in the Estimate of the Effect**
ModerateFurther research is likely to have an important impact on confidence in the estimate of the effect and may change the estimateLowFurther research is very likely to have an important impactVery lowAny estimate of effect is very uncertain

## 3. Results

After duplicates were removed, 278 studies were identified following the literature review. After reviewing titles and abstracts, 229 additional records were excluded: 39 review articles, two guidelines, nine studies including only participants with peculiar syndromes (Prader Willi, Down syndrome), 168 studies reporting outcomes different from those of interest, eight studies not available as full papers, three studies with a publication period before 2013, and one study with less than 10 patients.

**Figure 1:**
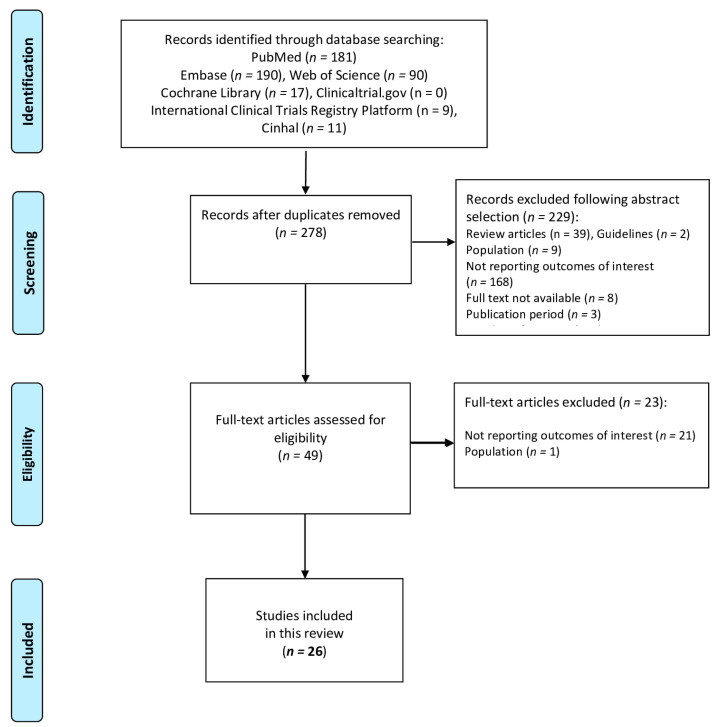
The PRISMA flow diagram.

A total of 49 full-text manuscripts were assessed for eligibility. After a full-text examination, 23 studies were excluded, and a final number of 26 studies were included in this systematic review. The PRISMA flow diagram (Figure 1) summarizes the study screening process. A summary of the studies included in this systematic review and the grading of evidence is reported in Table 1 [16,17,18,19,20,21,22,23,24,25,26,27,28,29,30,31,32,33,34,35,36,37,38,39,40].

Studies with a sample size of <50 patients or with a short follow-up (<6 months) have been considered at risk of bias in the study design and/or at imprecision for estimating the analyzed outcome. Therefore, they were assigned a low level of evidence. In the same way, other studies, retrospective in design or prospective without a control group, were evaluated in the entire design, considering sample size, follow-up range (a wide range makes comparisons less reliable), and outcome results. In some cases, the level of certainty was considered “low”. Below, we report the results on growth outcomes based on the 14 studies, graded as a moderate–high quality level (Table 2). None of these studies evaluated body composition or hormonal changes. Therefore, we report available evidence of low-quality grades.

### 3.1. Changes in Growth Pattern after Tonsillectomy

Among growth pattern outcomes, all the studies included in this review evaluated BMI z-score pre- and post-tonsillectomy (T) or TA; five studies analyzed the impact on height and/or height z-score, one on waist circumference [40], and in two studies, changes in the percentage of subjects with overweight/obesity were reported (Table 2). Below, we write a summary of the evidence.

#### 3.1.1. BMI z-Score

The baseline BMI z-score was reported as normal in three studies [17,37,40]. At the same time, in the other 11, the population was classified according to percentiles in two or more among these classes: underweight (Uw), normal weight (Nw), overweight (Ow), and obese (Ob); for these studies, the postoperative data were analyzed accordingly [16,22,23,24,25,27,32,33,35,36,39]. Two studies analyzed data from the same cohort of an RCT named Childhood Adenotonsillectomy trial (CHAT) [35,36].

BMI z-score did not change significantly compared to the control group in the two prospective studies in which participants were normal weight at baseline [17,37] in 1 RCT [40], and the 2 RCTs referred to the re-analysis of data of the CHAT cohort [35,36] (Table 2). In one large population-based cohort study, shortly after TA, the BMI z-score increased significantly in the operation group (0.41 ± 0.02) vs. the control group (0.18 ± 0.01; *p* < 0.001) [23]. In four studies that included subjects Uw, Nw, Ow, and Ob [22,24,27,32], BMI z-score increased across all cohorts, but the determinant for this result was the data of the Uw group, which was not the most prevalent (3.5%, 9.2%, 14.9%, 1.3%, respectively). Three studies, mainly involving patients Ow or Ob at baseline, reported an increased BMI z-score after TA [16,25,33]. A recent RCT in children with OSA concluded that there is an increase in BMI z-score between 0 and 12 months but not from 12–24 months, and the mean BMI z-score included catch-up of Uw subjects, while only eight of the TA group had undesirable weight gain [39].

Given the differences in ethnic origin, growth chart used for percentiles, and follow-up timing recorded in the various papers, a meta-analysis using BMI z-score as the outcome was not conducted. At the same time, we performed an analysis to search for determinants of BMI z-score.

#### 3.1.2. Determinants of BMI z-Score after TA

Baseline BMI z-score: A correlation between baseline BMI z-score and BMI z-score after TA was found in seven studies. Reduced BMI z-score at baseline (Uw population) was correlated to an increase in BMI z-score after TA in five studies [22,24,27,32,35], but not in the other two that vice versa reported an increased BMI z-score after TA in Ow–Ob subjects at baseline [25,33].

Age: Younger age (age < 4–6 years) was correlated to an increase in BMI z-score after TA in three studies [16,24,32] but not in the other two [22,33]; weight gain duration after TA: among the studies that reported an increase in BMI z-score, a rise between 0 and 12 months but not from 12–24 months was reported in one RCT study [39]. The other two studies reported increased BMI z-scores up to 24 months [22,33]. Most weight gain has been reported in the first six months after TA and only plateau one year after surgery in another study [32].

Surgery indication: Obstruction or OSA as an indication for TA, compared to recurrent upper airway infections, was correlated to an increase in BMI z-score after TA in one study [24] but not in the other three that evaluated this risk factor [16,22,33].

Gender: No significant difference was noted in BMI z-score between boys and girls in either the aggregate or subgroup analysis in the studies that analyzed this parameter [16,22,32,33,35].

Ethnicity: African-American children were noted to have significantly more weight gain increase than white or Hispanic and Asian children in one study [32], but these data were not confirmed in the other two studies [16,33].

Caloric intake: Increased caloric intake after TA was reported only in one study, classified as a low level of evidence [13], and none investigated physical activity levels. HT z-score increased significantly more than 12 months after TA, compared to the control group in three studies [23,24,33] but not in the other two after 18–24 months [32,39] (Table 2).

#### 3.1.3. Waist Circumference

Waist circumference was evaluated in only one study and did not change significantly after TA during the follow-up period of about ten months [40].

#### 3.1.4. Percentage of Overweight/Obesity

In most studies, an increase in the prevalence of Ow or Ob was not reported, as the increase in BMI z-score was minimal or absent during the follow-up period. In one study, children with Ow/Ob decreased in BMI z-score [22], whereas, in another, the Ow group had a 27% (6/22) shift to Ob [27] (Table 2).

### 3.2. Changes in Body Composition after Tonsillectomy

Only one study was selected for this systematic review and classified as low evidence. It showed a significant increase in body muscle mass after TA [18]. No study reported data on fat mass.

### 3.3. Hormonal Changes Influencing Post-Operative Growth after Tonsillectomy

Only three studies, classified as low level of evidence, reported data on hormonal changes, and below, we report a summary of the results [20,29,30] (Table 3).

IGF-1: The three studies measured IGF-1 levels pre-op and post-operation. IGF-1 levels increased significantly post-op vs. pre-op (*p* < 0.001) in two studies [29,30], whereas in the other study, only the obstructive group showed an increase in IGF-1 (*p* < 0.0001), but not the infection group (*p* = 0.0883) [20].

IGF-BP3: IGF-BP3 levels at 1-year post-op were significantly increased vs. pre-op (*p* = 0.001) in the only study that measured this hormone [29].

Ghrelin: Ghrelin levels increased significantly post-op vs. pre-op (*p* < 0.001), as reported in one study [30] and decreased 1-year post-op, as shown in another study (*p* < 0.001) [29].

Leptin: Pre-op leptin levels were significantly higher in patients than in controls (*p* < 0.001) and increased significantly post-op (*p* = 0.036), as reported in only one study [29].

## 4. Discussion

This systematic review selected 26 studies, considering 14 with moderately high-quality evidence. This allowed us to conclude that children who underwent TA could present an increase in BMI z-score. However, this weight gain is considered catch-up growth in subjects that are Uw at baseline, while normal weight or overweight TA did not lead to overweight per se. These data must be contextualized in an obesogenic environment where overweight subjects in the short term could gain more weight percentiles if they do not change their lifestyle [16,25,33,41,42], and studies with randomized controlled design consented to the separation of treatment effect from natural history [35,36,39,40].

The most recent systematic review on TA as a risk factor for childhood obesity was published in 2016 by Van M et al. and included six studies in children with OSA [9]; among these studies, four showed a significant weight gain after TA, and the others did not. Most of the included studies were observational and involved relatively few patients. One RCT, conducted by Katz et al., obtained in that review the greatest level of evidence [34], but in 2021 it was revised in the statistical analysis leading to the opposite conclusion [35,36]. At that time, Van M et al. concluded an association between TA and weight gain in patients with OSA in the short term [9]. This conclusion was the same reported in 2011 in a previous systematic review by Jeyakumar et al. who included nine studies and suggested a correlation between TA and weight gain in normal weight and overweight children, with a mean BMI increase of 7% [8]. They suggested large RCTs not available then to answer some of their questions [8].

In our systematic review, we attributed the highest level of evidence to the RCTs, even if GRADE classification is outcome-specific and RCTs do not need to have a high quality of evidence for the particular outcome we decided to analyze; however, the RCTs that we selected were well designed to answer our questions, and according to us, they add quality to the results of this systematic review. Notably, the children in the no-intervention group in these trials are not healthy ones but rather those who have OSA, a condition that appears to restrict their growth [9]. One RCT, conducted by Kevat et al., reported increased BMI z-score in the 12 months after TA, but not after, reporting a catch-up growth in Uw subjects, while undesirable weight gain was experienced only by eight participants (17% of the TA group) [39]. Another RCT showed that the BMI z-score remained similar at baseline and follow-up for both groups, and waist circumference did not increase in the TA group [40].

In two studies, Jensen et al. and Kirkham et al. presented a reanalysis of a multicenter randomized controlled trial (CHAT) comparing watchful waiting to early TA in 464 pre-pubertal OSA children between 2008 and 2011, with a 7-month follow-up [35,36]. This trial did not demonstrate differences in unwanted weight gain seven months after TA versus watchful waiting. Children in both arms experienced undesirable weight gain over seven months but did so similarly in the two arms (45% adenotonsillectomy vs. 41% watchful waiting; *p* = 0.400) [35,36]. A first analysis performed by Katz et al. in 2014 showed more significant weight increases seven months after TA in all weight categories, with children who failed to thrive that TA normalized weight but with increased risk for obesity in overweight children [34]. Years later, Jensen et al. and Kirkham et al. repeated the statistical analysis, and after excluding the thinnest children based on BMI < 20th percentile, regression models with a sensitivity analysis that excluded children who were underweight at baseline no longer reported a significant effect of TA on BMI z-score [35,36].

In our systematic review, there was heterogeneity in the outcomes of the ten prospective or retrospective studies that we rated as having moderate quality of evidence, primarily due to variations in the population covered and the study design. Firstly, there were differences in the study population enrolled in the studies regarding BMI percentile before surgery. The percentage of patients categorized by BMI percentile as Uw was not high (1.3–14.9%) in the studies that reported these data [22,24,27,32]; however, after TA, they showed a catch-up growth that influenced the mean BMI z-score of the whole cohort (as demonstrated by correlation analysis). We emphasize the fact that if 4.5% of Uw in the CHAT RCT were not excluded in the regression model, they were able to show or not a significant effect of TA on BMI z-score [35]. Secondly, considering the study design, the sample size was fundamental in these studies to determine whether the BMI z-score comparison was statistically significant. In the study of Ha EK et al. [23], the BMI z-score after TA of 0.41 ± 0.02 compared to 0.18 ± 0.01 in the control group was significant in this large Korean population study on 3172 children with TA and 31,663 controls, but this could have had no clinical impact at the individual level [23]. Vice versa, other studies included in this review presented sample sizes in the low hundreds in total and were then divided among the various weight categories; therefore, the study could be underpowered for some conclusions, and results should be viewed with caution [25]. The length of follow-up and the national growth charts used for BMI percentiles in various nations are two additional differences that may impact direct comparisons of study results.

The second outcome of this systematic review was identifying subgroups of children and adolescents at risk of weight gain. Not all the studies with moderately high levels of evidence were concordant, but different studies revealted that those who were underweight at baseline [22,24,27,32,35,36] or younger than 4–6 years [16,24,32] gained more weight after TA. Weight gain in children with failure-to-thrive after TA has been previously reported [43]. Age at risk is important to consider in clinical practice, as greater preoperative counseling, closer follow-up, and extra measures might be required for these young patients receiving TA to manage their obesity. Otolaryngologists and primary care providers should counsel families that increased weight gain could be possible in the first 6–12 months after TA, particularly in those under 6 years old [32]. In different cohort studies not included in this systematic review, a higher BMI pre-tonsillectomy was reported and associated with a history of repeated use of antibiotics, which probably affected weight by altering gut microbiota [44,45,46].

As a third outcome, we aimed to elucidate mechanisms beyond increased caloric intake that underlie weight gain. This was not possible due to the absence of caloric intake and physical activity reports before and after TA in most of the studies included in this review. Only one study that we had, classified as low-quality level of evidence, explored this outcome [13]. Dietary and physical records could help determine whether weight gain is caused by surgery or by parents who support specific recovery practices (overfeeding and restricting physical activity), and they could determine whether there is more to the story (i.e., hormonal, and metabolic changes), which could be intriguing for future research directions.

Previous studies, not included in this review because of their publication date being before 2013 and/or their failure to report weight variation within 24 months, showed conflicting results pre- and post-TA. In these studies, authors used diet records. Even though they are not a precise measurement of food intake, they are a practical and frequently used method of assessing caloric intake, type of food eaten, and the source of calories, and they have been shown to provide reliable assessments of food intake in children [12].

As previously reported, the surgical removal of tonsils might also improve the gag reflex and, therefore, eating patterns [47,48]. Rarely, adenoids will regrow after adenoidectomy, above all in young children, which could cause a plateau in weight gain [49,50], and the prevalence has been reported to be 8% [49].

A few studies compared children whose tonsils were removed and those who retained their tonsils. TA was not associated with higher levels of caloric intake and weight gain according to diet records reported in one of these studies [6]. In other studies on prepubertal children, significant weight gain associated with decreased mean caloric expenditure was reported [51]. In the latter study, the composition of the diet was similar in both groups, with no significant differences in the percentage of calories derived from protein, carbohydrates, or fat [51]. 

A few studies compared caloric intake pre- and post-TA in prepubertal children and found an increased caloric intake after TA. Gkouskou et al. found that, pre-operatively, the total calories received by children with tonsillar hypertrophy did not differ significantly from the control group. Six months after TA, they increased the calories they consumed daily, taking in even more significant amounts of food than the control group, resulting in significant weight gain without leading to overweight (catch-up growth) [52]. Selimoglu et al. found that, pre-operatively, healthy children had higher mean energy intake, weight, and height z-scores than children with adenotonsillar hypertrophy, and mean energy intake, weight, and height significantly increased after TA, again without leading to overweight [53]. Nachalon et al., in the study that we included in this review, reported increased caloric intake, weight, and height z-scores (without leading to overweight) after TA, as assessed by the Short Food Frequency Questionnaire (SFFQ), before and after TA, without considering a control group [13].

Regarding the diet composition reported in these studies, Gkouskou et al. found that the percentages of calories received from consuming sugar products, soft drinks, and edible fats were greater in children who had TA than in the control group. In contrast, the total calories received from other foods (meat, vegetables, or legumes) did not differ between children with TA and the control group [52]. The authors interpreted these differences as being due to parents’ recovery practices: they detected a possible inadequate caloric intake, so they provided the rest of the calories in soft drinks and candies as a more accessible and more pleasant form of food to consume [52]. Selimoglu et al. analyzed protein intake, and it was not statistically different in healthy children compared to children with adenotonsillar hypertrophy before and after TA [53]. Nachalon et al. reported a rise of 2% in protein and a decline of 4.49% in fat consumption. Although these changes were found to be significant, their actual effect on growth patterns was not apparent [13].

All these data suggest an increased caloric intake in children after TA as a possible mechanism underlying catch-up weight gain. Still, data on diet composition in children after TA are conflicting, and dietary interviews in the first few months after TA could elucidate this aspect. Furthermore, adenoids and tonsils represent vital constituents of the immune system, carrying profound implications also for immunology, physiology, and the microbiome [48]. The intricate interplay between adenoids, tonsils, and various aspects of human health has been reported [48], and their removal or reduction via surgery can profoundly affect growth through different pathways [48].

The main strengths of this review are the inclusion of all subjects who underwent tonsillectomy (not only for OSA), making our data transferable to the general population; a significant period of observation (10 years); the application of the PICOS model for studies’ selection and the GRADE system for assessment of evidence; and the presence of RCTs on the topic. Limitations are the heterogeneity of the analyzed studies reported above regarding the population analyzed and study design.

## 5. Conclusions

Our findings suggest that TA may not be an independent risk factor for unfavorable weight gain in children undergoing TA. RCT studies support this evidence by distinguishing the effect of TA from the natural history in an obesogenic context. However, children who were Uw pre-operatively or younger than six years are reported as gaining more weight after TA than expected. Therefore, otolaryngologists and primary care providers should counsel families that increased weight gain could occur in the first 6–12 months after TA, particularly in those under six.

This systematic review highlights the need for further studies, including caloric intake reports, to better understand the mechanisms underlying weight gain in these categories of patients and to ascertain a possible role of microbiome change with tonsillectomy.

## Figures and Tables

**Table 1:** Literature analysis after PICOS selection: summary of the studies and grading of evidence by the GRADE system.

References	Main Objective	Study Design	Population and Comparator	Methods	Outcomes	Results	Study Limitations and Level of Evidence
Smith DF et al. [16]	Post-op WT and demographic risk factors	Retrospective	115 children with TA (85 had surgery for OSA; 30 for RT) Pre-op BMI z-score: 0.98 ± 1.5 Age: 1–18 y (7.2 ± 4.3 y) Follow-up: 6 m Period: 2008–2011 Region: USA	Chart review	HT, WT, BMIz changes: pre-op vs. post-op Risk factors: age, gender, race	BMIz increased from 0.98 ± 1.5 to 1.21 ± 1.25 (*p* = 0.0009) No difference by surgical indication, gender, race Younger age (<6 years) was a significant predictor of postoperative weight gain (*p* = 0.015)	Retrospective Moderate
Huang YS et al. [17]	Efficacy of TA in children with OSA	Prospective	88 children with OSA underwent TA Pre-op BMI z-score: −0.7 ± 1.0 Age: 6–12 y (8.9 ± 2.7 y) Follow-up: 36 m Period: 2007–2010 Region: Taiwan	Medical history, physical examination	BMIz pre-op vs. post-op	BMIz increased not significantly from −0.7 ± 1.0 to −0.33 ± 0.98 at 24 m (*p* > 0.05)	No control group Selection bias (no Obese by choice) Moderate
Koycu A et al. [18]	Effect of TA on muscle and fat composition	Prospective	30 prepubertal children with TA or A only vs. 28 controls Pre-op BMIz 0.09 ± 1.09 Age: 3–9 y (5.0 ± 1.2) Follow-up: 6 m Period: 2013–2014 Region: Turkey	Questionnaire at 0 and 6 m post-op Growth and body composition data at 0 and 6 m with bioelectrical impedance analysis (BIA)	Dietary habits and physical activity HT and WT z-scores, body fat and muscle mass, BMI z-scores, basal metabolic scores	At 6 m, in both groups: increased body muscle mass vs. baseline (*p* < 0.05) Relative BMIz (relBMI), pre-post intervention, increased significantly (*p* = 0.018) only in the patient group No change (pre–post) in body fat mass, body fat %, HT z-scores, WT z-scores, and BMIz Children with TA (n = 14): BMIz improved significantly (*p* < 0.05), whereas children who underwent A alone (n = 16) had no significant change in BMI after surgery The number of OW and Ob children did not change significantly in either group (*p* < 0.05) BMIz were similar at the end of 6 m in the three groups	Small sample size Low
Inja RR et al. [19]	Effect of T on swallowing	Prospective	29 with TA and 2 T only Pre-op WT: 25.4 ± 12.4 Kg Age: 4–14 y (8.3 ± 2.9) Follow-up: 3 m Period: not reported Region: South India	WT at 0, 1 and 3 m post-op	WT	The increase in WT post-op (+3 m) was 26.9 ± 12.7 (vs. pre-op visit, *p*-value < 0.001)	Small sample size Short f/up No control group HT and BMI and z-scores not evaluated. Low
Elnashar I et al. [20]	Effect of T on the serum IGF-1 in patients undergoing T	Prospective	50 children with T (24 because of obstructive symptoms and 25 because of RT) Pre-Op BMI: n.a. Age: 4.5–10 y Follow-up: 3 m Period: 2013–2014 Region: Egypt	IGF-1 pre-op and 3 m post-op	IGF-1 levels	In the obstructive group, there was an increase in IGF-1 (*p* < 0.0001) but not in the infection group (*p* = 0.0883) The pre-op IGF-1 levels in the obstructive group was less than that in the infective group (*p* = 0.026), whereas the post-op IGF-1 level was higher in the obstructive group than in the infective group (0.01)	Short f/up No control group HT, WT, BMI not evaluated. Low
Costa Pinto R et al. [21]	Effect of TA on WT and HT	Retrospective	29 children with TA Pre-op WT 29.1 ± 17.4 kg, HT 122 ± 27.1 cm Age: 3–13 y Follow-up: 4 months Period: not reported Region: Brazil	WT and HT pre-op and 4 m post-op	WT and HT	WT: from 29.1 ± 17.4 kg to 32.8 ± 18.7 kg (*p* < 0.0001). HT: from 122 ± 27.1 cm to 125 ± 25.4 (*p* < 0.0001) Pre-scholar, Scholar, Adolescent age, WT and HT increases are not significant.	Small sample size Short f/up Retrospective No z-score Low
Beauchamp MT et al. [22]	Effect of T or TA on BMIz	Retrospective	1751 children with T or TA Age 3–11 y Groups for WT: (383 Ob, 266 Ow, 1041 Nw and 61 Uw) Groups for age: “young” (ages 3–5 y) and “older” (ages 6–11 y) Follow-up: 24 m Period: 2004–2017 Region: USA	Data from medical records	Change in BMIz trajectories pre-op vs. post-op Predictors: age at time of surgery and gender	Age at time of surgery and gender were not significant predictors of BMIz change Children with Ow/Ob decreased in BMIz, whereas children with Uw or Nw experienced an increase in BMIz.	Retrospective Wide range period Moderate
Ha EK et al. [23]	Effect of TA on BMIz and HTz	Prospective	3172 children with TA and 31,663 in the control group Age: 34–69 m Follow-up: 66–71 m Period: 2008–2009 Region: Korea	Physical measurement by a primary clinician	BMI, WT and HT	TA was related to increased WT and HT in the operation group vs. the control group. Shortly after TA, WT and BMIz increased significantly in the operation group (0.41 ± 0.02) vs. control group (0.18 ± 0.01; *p* < 0.001); a significant increase in the HT z-score was observed more than 1 y after TA.	Moderate
Han SC et al. [24]	Effect of TA on change in growth-for-age	Retrospective	206 prepubertal children with TA; 167 Nw; 19 Uw; and 20 Ob Age: 3–10 y Follow-up: 12 m Period: 2011–2014 Region: Seoul	OSA-18 questionnaire, symptom questionnaire. Data from medical records	Change in HT, WT, BMIz pre-op vs. post-op Factors affecting the change in growth after TA	After TA, HTz, WTz, and BMIz all increased both in 167 Nw patients and 19 Uw patients (*p*-value < 0.05). In 20 Ob patients, only HTz increased (*p* value = 0.028). The multiple regression test showed that the sleep disturbance domain of OSA-18 was positively correlated with HT z-score change (*p*-value = 0.041), and age was negatively correlated with WT z-score change (*p* value = 0.016). Pre-op BMIz was negatively correlated (*p*-value = 0.019) and adenoid grade was positively correlated (*p*-value = 0.023) with BMIz change.	Retrospective Small number of patients in the Uw and Ob group Moderate
Kim JY et al. [25]	Effect of TA on behavior in children with sleep-disordered breathing (SDB)	Prospective	170 children who underwent TA and 150 controls BMI pc: 57.1 ± 30.7 Age 5–11 y Follow-up: 15.4 ± 2.7 m Period: 2013–2014 Region: South Korea	Standardized questionnaires for parental sleep-related breathing disorder (SRBD); clinical assessments	Change in HT, WT, BMI pc	Significant increase in BMI pc (*p* < 0.001) and percentage of Ow (*p* < 0.05) after surgery compared to controls	Moderate
Voora RS et al. [26]	Impact of T on obesity in Pediatric Patients With SDB	Retrospective	560 (of the cohort of 1153) had available follow-up BMI data. They underwent T or TA due to SDB (87.8%) or (12.2%) for recurrent tonsillitis (RT). Age: 2.0–19.5 y (7.6 ± 4.0 y) Follow-up: 6 m (50–605 d) Period: 2016–2017 Region: USA	HT, WT, BMI pre-op and post-op	Change in BMI pc pre-op vs. post-op	Higher post-op BMI pc Post-op BMI pc strongly correlated to higher pre-op BMI pc (*p* < 0.001), as well as younger age (*p* < 0.001), male sex (*p* = 0.0005), and SDB as a surgical indication (*p* = 0.003).	Retrospective Limited % of patients with available follow-up BMI data Wide f/up period Low
Hsu WC et al. [27]	Impacts of WT status on surgical outcomes and shifts of WT status after TA in children with OSA	Prospective	161 children with OSA who underwent TA. Children were divided into four WT status groups (24 Uw, 79 Nw, 22 Ow and 36 Ob), based on age- and gender-corrected BMI Age: 7.0 ± 3.4 y Follow-up: 6 m Period: 2009–2011 Region: Taiwan	Demographic data, clinical symptoms, and physical examinations	Change in WT pre-op vs. post-op	54% (13/24) of the Uw children gained WT and shifted to Nw. Nw group had a 9% (7/79) shift to Ow status and 4% (3/79) shift to Uw. The Ow group had a 27% (6/22) shift to Ob and a 14% (3/22) shift to Nw status. Most Ob children (92%, 33/36) remained Ob after TA, with only 8% (3/36) shifting to Ow status	No control group Moderate
Koren D et al. [28]	Impact of TA upon Ob and metabolic outcomes in children with OSA	Retrospective	69 children with moderate-severe baseline OSA who underwent TA Age: 6.33 ± 2.04 y (3.8–12.2) Follow-up: 7.9 ± 3.1 m (range, 2.2–12.2 m) Period: n.a. Region: USA	Baseline and at follow-up, overnight PSG anthropometric and metabolic measurements	BMIz, insulin, glucose, HOMA-IR, McAuley index, total-HDL-LDL cholesterol, triglycerides	BMIz increased significantly (from 1.43 ± 0.78 to 1.52 ± 0.62, *p* < 0.001) whereas fasting plasma insulin and HOMA-IR were significantly lower and McAuley index trended toward being higher, indicating improved insulin resistance. HDL was significantly higher and total cholesterol/HDL and LDL/HDL ratios were significantly lower, indicating more favorable lipoprotein profiles.	Retrospective Short follow up per some patients Wide age distribution and range of f/up Low
Karalok ZS et al. [29]	Impact of T or TA on serum leptin and plasma ghrelin levels in children	Prospective	31 patients with T or TA 29 age and sex-matched healthy control children Age: 5.70 ± 2.37 y Follow-up: 12 m Period: 2010 Region: Turkey	Auxologic evaluation and biochemical investigations	WT, HT, BMI, IGF-1, IGFBP-3, HOMA-IR, leptin, and ghrelin	1 y post-op, HTz (*p* = 0.001) and WTz (*p* = 0.004) were significantly increased in both groups. No changes in BMIz (*p* = 0.105) were observed Pre-op leptin levels were significantly higher in patients than controls (*p* < 0.001). IGF-1, IGFBP-3, HOMA-IR, and ghrelin values were not significantly different between the groups. 1-year post-op, IGF-1 (*p* = 0.001) and IGFBP-3 (*p* = 0.001) were significantly increased, while ghrelin (*p* < 0.001) was significantly decreased; leptin levels were also significantly higher than pre-op values (*p* = 0.036).	Small sample size Low
Moghaddam YJ et al. [30]	Impact of TA on IGF-1 and ghrelin in children	Prospective	40 pre-pubertal children with AT hypertrophy, SDB, snoring, open mouth breathing and growth retardation Age: 6.57 ± 1.28 y Follow-up: 12 m Period: 2010–2011 Region: Iran	Auxologic evaluation and biochemical investigations	WT, HT, BMI percentiles, IGF-1, ghrelin	WT, HT, and BMI percentiles were increased significantly post-op (*p* < 0.001). Serum IGF-1 and ghrelin levels increased significantly post-op vs. pre-op (*p* < 0.001).	Small sample size No control group Low
S YB et al. [31]	Influence of TA on WT	Prospective	45 children divided in 2 groups: 28 (5–10 y) 17 (11–15 y) Mean age 9.32 y ± n.a Follow-up: 6 m Period: n.a. Region: India	Growth data	Change in HT, WT pre and post op	No significant change in WT between two groups and between pre-op and post-op phasis	Small sample size No control group Low
Czechowicz et al. [32]	Changes in WT, HT, BMIz, before and after TA	Retrospective	815 children with TA Pre-op WT pc 1st–60th: 342 pt 61st–80th 142 pt 81st–99th 259 pt <1st 11 pt, >99th 61 pt Pre-op BMI z-score: n.a. Age: <18 y <4 y: 206 4–8 y: 338 >8 y: 199 Follow-up: 18 m Period: 2007–2012 Region: Stanford, California	Pre- and post-op WT and HT	HT, WT, BMIz changes: pre-op vs. post-op	Mean WT pc increased by 6.3 pc points (*p* < 0.001). No significant changes in HT pc. Weight percentile plateaued 1 year after surgery, with 90% of gain occurring by 41⁄2 to 6 months. Mean BMI pc increased from 64 to 72 (*p* < 0.001) Lower BMI at the time of surgery (1st–80th pc) was a significant predictor of post-op WT gain (*p* < 0.001); no significant increase in BMI pc in those with higher BMI (81st–99th). African-American children had a higher WT pc increase (*p* < 0.001). Tendency to greatest increases in WT were in the younger population (<4 y: mean increase of 10.7 points; children > 8 years had a smaller increase: 3.8 points).	Retrospective Moderate
Lewis TL et al. [33]	Association between WT gain and TA	Retrospective	154 children with TA; 182 as the control group Study populations divided into 3 groups: Nw (BMI < 85 °C), Ow (85–95 °C), Ob (>95 °C) Pre-op BMI pc: Nw 45.7, OW 90, Ob 96.7 Age: 2–16 y (mean: Nw 4.9, Ow 6.6, Ob 8.3) Follow-up: 6–24 months Period: 2010–2011 Region: USA	Pre- and post-op WT, HT, BMIz	WT, HT, BMIz Risk factors: Baseline WT, age, gender, ethnicity, indication for surgery	WT gain in the TA group significantly increased at 6, 12, 18, 24 months (*p* 0.01, <0.001, <0.001, 0.002, respectively) HT changes were significantly different at 24 months (TA + 1.8 cm, *p* 0.04) Ob: significant WT gain at 12, 18, 24 m (*p* < 0.001); significant increases in HT at 6–12 m Nw: significant WT gain at 12 months (*p* 0.02) Ow: no differences BMIz significantly higher in Ob at 24 m Differences not affected by age, gender, ethnicity, indication for surgery	Retrospective Incomplete follow-up data Nw in TA group significantly younger than controls Moderate
Katz ES et al. [34]	WT gain post-TA	RCT (CHAT)	396: 204 children with OSA underwent TA; 192 watchful waiting control group Pre-op BMI pc: Nw: 48.3%, Ow: 14.9% Ob: 32.7% Uw: 4.5% Age: 5–9.9 y (6.57 ± 1.43 in the TA group) Follow-up: 7 m Period: 2008–2011 Region: USA	Pre- and post-op WT, HT, BMIz	WT, HT, BMIz Risk factors: Baseline WT, age, gender, ethnicity, indication for surgery	WTz and BMIz increases (0.13 vs. 0.31) in both groups, greater with TA (*p* < 0.0001) A greater proportion of Ow randomized to TA developed Ob compared with controls (52% vs. 21%; *p* = 0.05) Race and gender were not significantly associated with BMIz change	Statistical model did not consider variability of baseline WT status (Kirkham et al.) Low
Jensen AM et al. [35]	Determine if TA increases the risk of Ob	Re-analysis of RCT (CHAT)	396: 204 children with OSA underwent TA; 192 watchful waiting control group Pre-op BMI pc: Nw: 48.3%, Ow: 14.9% Ob: 32.7% Uw: 4.5% Age: 5–9.9 y (6.57 ± 1.43 in the TA group) Follow-up: 7 m Period: 2008–2011 Region: USA	Pre- and post-op %BMI pc 95 (as a percentage of the 95th percentile)	%BMI pc 95 Baseline WT, Age, sex, race, Sleep apnea	Utilizing a linear mixed-effects model accounting for the variability of baseline weight status, OSA resolution status, and time, no significant association between AT and %BMIp95 was found WT significantly increased in the TA group, especially in Uw at baseline (*p* = 0.01). No gender difference. Resolution of OSA was associated with a decreased weight trajectory (*p* < 0.001)	High
Kirkham et al. [36]	Effect of TA on BMIz Factors influencing post-op growth	Reanalysis of RCT (CHAT) CHAT: children 5–9.9 y with OSA (proven by PSG)	396: 204 children with OSA underwent TA 192 watchful waiting control group Pre-op BMI pc: Nw: 48.3%, Ow: 14.9% Ob: 32.7% Uw: 4.5% Age: 5–9.9 y (6.57 ± 1.43 y in the TA group) Follow-up: 7 m Period: 2008–2011 Region: USA	Pre- and post-op BMIz	WT, HT, BMIz Baseline WT, Age, Sleep apnea, maternal BMI	A similar percentage of children in both arms experienced undesirable WT gain (45% TA vs. 41% watchful waiting). Regression models excluded children who were Uw at baseline; there was no longer a significant effect of TA on BMIz (different from Katz 2014).	High
Al Abdulla A et al. [37]	WT gain post-TA	Retrospective	240 children Age: 1–15 (7.45 ± 2.89 y) Pre-operative BMI 22.35 ± 3.98 kg/m^2^ Follow-up: 6 m Period: 2018–2019 Region: Saudi Arabia	Pre- and post-op BMI	WT, HT, BMI, and classification in Uw, Nw, Ow, Ob according to percentile cut-off	Significant differences in mean WT and BMI at 1–6 months post-op (*p* < 0.0001) Positive linear correlation between WT and BMI at 6 m	Retrospective No z-scores calculation Low
AlAbdullah ZA et al. [38]	Impact of TA on growth	Prospective	53 children underwent T. 52 controls Age: 2–14 y Pre-operative BMIz −0.30 ± 2.06 Follow-up: 12 m Period: 2012–2015 Region: Saudi Arabia	Pre- and post-op BMIz	WT, HT, BMI, BMIz	BMIz in the study group increased compared to controls, but this gain was not statistically significant (*p* = 0.053).	Moderate
Kevat A et al. [39]	Impact of TA on growth trajectories in children with OSA	RCT	126 children with OSA randomly assigned to early TA (within 2 m) routine (12 m) Age: 49.46 ± 8.44 m 48.97 ± 8.23 m pre-operative BMIz 0.22 (−0.43–0.97) BMIz 0.56 (−0.24–1.21) Follow-up: 2 y Period: 2018–2019 Region: Australia	BMIz pre-op and at 12–24 m	WTz, HTz, BMIz	Significant increase in BMIz in the 12 months after TA. Early surgery group (0–12 m): median 0.4, IC95 0.1–0.8. Routine surgery group (12–24 m) median 0.45, IC 95 0.1–0.8, but not from 0–12 months (preoperative time). Findings for WTz were like the findings for BMIz. HTz: no significant change between different time points or intervention groups.	High
Nachalon Y et al. [13]	Impact of TA on growth in children with OSA	Prospective	16 children with OSA Age: 23 ± 6 m Follow-up: 5 ± 2 m Period: 2013 Region: Israel	Pre- and post-op WT, HT, BMIz Caloric intake questionnaire	HT, WT, C-reactive protein, IGF-1	Increase in HT, WT (*p* < 0.001 for both) WTz, BMI and BMIz (*p* = 0.002). Increased caloric intake after TA (*p* < 0.001) with increased protein and decreased fat intake. The decrease in C-reactive protein levels correlated with the increase in WT in boys (*p* < 0.05, adjusted for caloric intake).	Small sample size No control group Low
Au CT et al. [40]	Impact of T or TA on growth	RCT	71 pre-pubertal children with OSA 35 with early surgical intervention (18TA, 17T) 36 watchful waiting Age: 6–11 y (8.4 ± 1.6) Follow-up: 10.5 ± 2.2 m Period: 2010–2018 Region: Hong Kong	Pre- and post-op WT, BMIz, waist circumference z-score	WT, BMIz, waist circumference z-score	The intervention group had a higher baseline waist circumference z-score (*p* = 0.063). The intervention group had a significantly greater increase in WT +3.3 ± 2.1 vs. +2.2 ± 1.5 kg, *p* = 0.014. The BMI z-score remained similar at baseline and follow-up for both groups. The waist circumference z-score increased significantly in the control group (*p* < 0.05).	Wide f/up range High

Abbreviations: adenotonsillectomy (TA), adenoidectomy (A), tonsillectomy (T), obstructive sleep apnea (OSA), recurrent tonsillitis (RT), pre-operative (pre-op), post-operative (post-op), polysomnography (PSG), height (HT), height z-score (HTz), weight (WT), weight z-score (WTz), body mass index (BMI), BMI z-score (BMIz), percentile (pc), Overweight (Ow), Obese (Ob), Normal weight (Nw), Underweight (Uw), n.a. not available, sleep-disordered breathing (SDB), day (d), month (m), year (y), Randomized controlled trial (RCT), Childhood Adenotonsillectomy trial (CHAT). Among the selected studies, 5 were RCTs, 12 were prospective, and nine were retrospective. The number of children enrolled in the studies who underwent tonsillectomy (with or without adenoidectomy) was between 16 and 3172: in eight (8) studies, participants were ≤50, in thirteen (13) studies, participants were between 51 and 300, and in five (5) studies, participants were more than 300. The follow-up period lasted from 3 to 36 months (we analyzed up to 24 months by design of the review).

**Table 2:** Summary of studies with moderate–high level quality of evidence.

Reference	Initial WT Status	Number/Design	Intervention (f/up)	WT	HT	BMIz	Risk Factors for WT/BMI Change	Level of Evidence (GRADE)
Smith DF et al. [16]	Nw-Ow	115/R	TA (6 m)			↑	<age (<6 y) No OSA indication	M
Huang YS et al. [17]	Nw	88/P	TA (36 m)			=		M
Beauchamp MT et al. [22]	Uw, Nw, Ow, Ob	1751/R	TA or T (24 m)			↑ in Uw/Nw ↓ in Ob-Ow	Uw No age, no surgery indication	M
Ha EK et al. [23]	Uw, Nw, Ow, Ob	3172/P	TA (66–71)	↑	↑ HTz	↑		M
Han SC et al. [24]	Nw, Ow, Ob	206/R	TA (12 m)	↑ WTz	↑ HTz	↑	<age <BMI pre >adenoid grade	M
Kim JY et al. [25]	Nw, Ow, Ob	170/P	TA (15 m)			↑ (and >Ow)	TA	M
Hsu WC et al. [27]	Uw, Nw, Ow, Ob	161/P	TA (6 m)			↑	Uw	M
Czechowicz et al. [32]	Uw, Nw, Ow, Ob	815/R	TA (18 m)	↑ WTz	=HTz	↑	<age (<4 y) <BMI pre African-American	M
Lewis TL et al. [33]	Nw, Ow, Ob	154/R	TA (24 m)	↑	↑	↑	No age Ob and Nw pre No surgery indication	M
Jensen AM et al. [35]	Uw, Nw, Ow, Ob	396/RCT	TA (7 m)	↑		=	Uw at baseline	H
Kirkham et al. [36]	Uw, Nw, Ow, Ob	396/RCT	TA (7 m)	=		=	Uw at baseline	H
AlAbdullah ZA et al. [38]	Nw	53/P	T (12 m)			=		M
Kevat A et al. [39]	Uw, Nw, Ow, Ob	126/RCT	TA (24 m)	↑	=HTz	↑		H
Au CT et al. [40]	Nw	71/RCT	T-TA (10 m)	↑		=		H

Uw: underweight, Nw: normal weight, Ow: overweight, Ob: obese, R: retrospective, P: prospective, RCT: randomized controlled trial, WT: weight, WTz: weight z-score, HT: height, HTz: height z-score, BMI: body mass index, BMIz: BMI z-score, OSA: obstructive sleep apnea, T: tonsillectomy, TA: adenotonsillectomy, M: moderate, H: high. ↓: reduced; ↑: increased; =: no variation.

**Table 3:** Summary of studies with low quality of evidence.

Reference	Intervention (f/up)	WT	HT	BMIz	relBMI%	Body Composition	Hormonal Change	Risk Factors for WT/BMI Change	Level of Evidence (GRADE)
Koycu A et al. [18]	TA or A (6 m) Nw	=	=		↑ relBMI% ↑ BMIz in TA	↑ body muscle mass		TA vs. T	L
Inja RR et al. [19]	TA or A (3 m)	↑							L
Elnashar I et al. [20]	T (3 m)						↑ IGF-1 in obstructive group, not in infective		L
Costa Pinto R et al. [21]	TA (4 m)	↑	↑						L
Voora RS et al. [26]	T or TA (6 m)			↑				↑ BMI pre-op Younger Male Obstruction as indication	
Koren D et al. [28]	TA (8 m)-OSA			↑					L
Karalok ZS et al. [29]	T or TA (12 m)	↑ WTz	↑ WTz	=			Pre-OP. ↑ leptin = GHrelin, IGF.1 +1 y post-op: ↑ IGF-1, IGFBP3, leptin and ↓ GHrelin		L
Moghaddam YJ et al. [30]	TA (12 m)-OSA	↑	↑	↑			Post-op: ↑ IGF-1, leptin, and Ghrelin		L
S YB et al. [31]	TA (6 m)	=							L
Katz ES et al. [34]	TA (7 m)-OSA Uw, Nw, Ow, Ob	↑ WTz	=	↑				No race and gender	L
Nachalon Y et al. [13]	TA (5 m)-OSA	↑/WTz	↑	↑				Increased caloric intake	L
Al Abdulla A et al. [37]	TA (6 m)	↑							L

Uw: underweight, Nw: normal weight, Ow: overweight, Ob: obese, WT: weight, WTz: weight z-score, HT: height, BMI: body mass index, BMIz: BMI z-score, relBMI%: relative BMI percentage, IGF-1: insulin like growth factor-1, pre-OP: pre-operation, OSA: obstructive sleep apnea, T: tonsillectomy, TA: adenotonsillectomy, M: moderate, H: high. ↓: reduced; ↑: increased; =: no variation.

## Data Availability

All databases generated for this study are included in the article.
